# Fructooligosaccharide Reduces Weanling Pig Diarrhea in Conjunction with Improving Intestinal Antioxidase Activity and Tight Junction Protein Expression

**DOI:** 10.3390/nu14030512

**Published:** 2022-01-25

**Authors:** Zeyu Zhang, Ge Zhang, Shuai Zhang, Jinbiao Zhao

**Affiliations:** State Key Laboratory of Animal Nutrition, College of Animal Science and Technology, China Agricultural University, Beijing 100193, China; mafic2017zeyu@163.com (Z.Z.); zhangge0557@163.com (G.Z.); zhangshuai16@cau.edu.cn (S.Z.)

**Keywords:** fructooligosaccharide, intestinal barrier, gut microbiota, antioxidant capacity, weanling pigs

## Abstract

This study was to illustrate the effects of fructooligosaccharide (FOS) on the antioxidant capacity, intestinal barrier function, and microbial community of weanling pigs. Results showed that FOS reduced the incidence of diarrhea (6.5 vs. 10.8%) of pigs (*p* < 0.05) but did not affect growth performance when compared with the control group. A diet supplemented with FOS increased ileal mRNA expression of occludin (1.7 vs. 1.0), claudin-1 (1.9 vs. 1.0), claudin-2 (1.8 vs. 1.0), and claudin-4 (1.7 vs. 1.0), as well as colonic mRNA expression of ZO-1 (1.6 vs. 1.0), claudin-1 (1.7 vs. 1.0), occludin (1.9 vs. 1.0), and pBD-1 (1.5 vs. 1.0) when compared with the control group (*p* < 0.05). FOS supplementation improved the anti-oxidase activity and expression of nuclear factor erythroid-2 related factor 2 (Nrf2), and decreased concentrations of D-lactate (3.05 U/L vs. 2.83 U/L) and TNF-α (59.1 pg/mL vs. 48.0 pg/mL) in the serum when compared with the control group (*p* < 0.05). In addition, FOS increased Sharpea, Megasphaera, and Bacillus populations in the gut when compared with the control group (*p* < 0.05). Association analysis indicated that mRNA expression of occludin and claudin-1 in the ileal mucosa were correlated positively with populations of Sharpea and Bacillus (*p* < 0.05). Furthermore, mRNA expression of occludin and claudin-1 in the colonic mucosa were correlated positively with abundances of Sharpea, Lactobocillus, and Bifidobacterium (*p* < 0.05). In conclusion, FOS activated Nrf2 signaling and increased the expression of specific tight junction proteins, which were associated with reduced diarrhea incidence.

## 1. Introduction

Fructooligosaccharide (FOS) as a functional oligosaccharide is fermented by gut microbiota to produce lactic acid and short chain fatty acids (SCFA), which play an important role in regulating host nutrition and health via activating G protein-coupled receptors (GPR) and inhibiting activity of histone deacetylase (HDAC) [1,2]. In addition, SCFA directly reduces a favorable intestinal pH and increases growth of Lactobacillus and Bifidobacterium in the foregut, leading to a dynamic balance of the gut microenvironment [3]. Previous researchers have reported that dietary supplementation of FOS in the last 4 weeks of gestation through to 4 weeks of lactation for sows accelerated the development of the intestinal immune system in offspring [4,5]. Dietary FOS treatment of new-born piglets for 2 weeks upregulated tight junction protein expression and increased microbial diversity in the colon, as well as promoted immune system development of piglets [6]. Many researchers have reported that FOS administration increases abundances of Lactobacillus and Bifidobactrium in order to produce more SCFA, which can activate GPR 43 and GPR 109A to suppress the signaling of NF-kB, leading to improved expression of host defense peptides and reduced pro-inflammatory cytokines [7,8]. However, actions of FOS on regulating the redox status of the host and the relationship between gut microbial community and intestinal barrier function shaped by FOS supplementation have been unclear.

Weaning stress results in damage of intestinal barrier function and unbalanced redox status in infants and young animals [9]. Normally, the stress of weaning leads to the increased concentration of reactive oxygen species to induce damage to structures and the physiological function of mitochondria in the epithelial cells, as well as imbalance of gut microbial composition, resulting in injury of the intestinal barrier function and altered gut permeability [10]. Considering positive responses of functional FOS on intestinal barrier function and microbial community composition, we hypothesized that FOS would relieve weaning stress and prevent weaning-induced damage of intestinal barrier function by regulating intestinal redox status. Therefore, the aim of this study was to understand the changes in antioxidant capacity, intestinal barrier function, and microbial community when weanling pigs were fed FOS.

## 2. Materials and Methods

### 2.1. Animals, Dietary Treatments and Experimental Design

Weanling pigs (*n* = 72) with an initial average body weight of 6.76 ± 0.22 kg and age of 24 ± 3 d were allocated into 2 dietary treatments in a completely randomized design. Dietary treatment included a control diet (NC) and the tested diet, containing 1% FOS. The FOS was synthesized from sucrose by using fructosyltransferase and plant active carbon with a purity of 90%, and it contained 4~6 of glycosidic bonds connecting residues of fructose. Each dietary treatment included 6 replicates (pens) and 6 weanling pigs (3 gilts and 3 barrows) per replicate. Pigs in each replicate were selected from different lactating sows to avoid maternal effects on this study. Composition of feed ingredient and dietary nutrient concentrations are shown in Supplementary Table S1. Piglets’ nutrient requirements for standardized ileal digestible amino acids, trace minerals, and vitamins were satisfied according to the recommendations of nutrient requirements of swine [11]. This experiment lasted 21 d. Pigs were provided *ad libitum* access to water and food. The humidity and temperature were controlled at 50~60% and 25~28 °C, respectively. On day 0 and day 21, the body weight of pigs and their feed consumed were weighed to calculate the average daily feed intake (ADFI) and average daily gain (ADG). In addition, the diarrhea incidence of pigs was recorded every day and estimated by each pen as the proportion of days in which pigs showed clinical symptoms, with respect to the total number of days of the trial, according to a previous study [12]. On the morning of day 21, one piglet, which was closest to the average body weight of the piglets in a same pen, was selected from each pen and euthanized to collect samples of intestinal tissue and digesta from the ileum and colon. Samples of blood were collected from one pig in each pen to separate the serum for further analysis.

### 2.2. Samples Collection

The samples were collected following the previous report [13]. Approximately 8 mL of blood samples from the euthanized pigs in each pen were collected into vacutainer tubes via jugular vena puncture. Then, about 1 mL of serum was collected after centrifugation at 3000× *g* for 15 min at 4 °C, and stored at −20 °C for further analysis. Middle intestinal segments (3 to 5 cm) of the ileum and colon were excised, rinsed with 0.9% saline solution to clean intestinal contents, and the samples were put into 4% paraformaldehyde to be fixed. Ileal and colonic mucosa were scraped using a microscope slide and the scrapings were placed in centrifuge tubes (2.5 mL). Mucosal samples were frozen using liquid nitrogen and stored at −80 °C for further analysis. In addition, ileal and colonic digesta were collected into centrifuge tubes (50 mL) and then stored at −80 °C.

### 2.3. Serum Parameters

Serum parameters, including antioxidant capacity and inflammatory responses, were analyzed [13]. Porcine enzyme-linked immunosorbent assay kits (Shanghai Enzyme-linked Biotechnology Co. Ltd., Shanghai, China) were applied to determine concentrations of serum D-lactate and diamine oxidase (DAO). The assay kits (Beijing Kangjia Bioengineering Company, Beijing, China) were used to analyze antioxidant capacity, including serum malondialdehyde (MDA), superoxide dismutase (SOD), and glutathione peroxidase (GSH-Px). The ELISA test kits (Groundwork Biotechnology Diagnostics Ltd., San Diego, CA, USA) were used to determine serum concentrations of interleukins, including IL-1β, IL-6, and IL-10. Serum content of tumor necrosis factor-α (TNF-α) was measured using a radioimmunoassay kit (North Institute of Biotechnology, Beijing, China).

### 2.4. Intestinal Morphology

Morphology of the intestinal tissues were analyzed using hematoxylin and eosin (H&E) staining, according to a previously described protocol [14]. Digital images of intestinal morphology at 10× magnification from the ileum and colon were obtained using a light microscope. In a digital image, villus height and crypt depth from 10 different fields of view through a microscope were recorded, and the average villus height and crypt depth were calculated.

### 2.5. mRNA Expression of Inflammatory Cytokines, Host Defense Peptides, Tight Junction Proteins

The RNA extraction, reverse transcription, and real-time PCR procedures were performed according to a previous study [15]. The GAPDH was used as the housekeeping gene. All primers were chosen to perform expression of genes that are related to inflammatory cytokines, host defense peptides, tight junction proteins, and mucins (Supplementary Table S2), and were purchased from Generay Company (Shanghai, China). The real-time polymerase chain reaction (PCR) results were analyzed using a 2^−^^ΔΔCt^ method. The mRNA levels of genes were expressed as the fold change relative to the mean value of the control diet.

### 2.6. Expression of NF-κB and Nrf2 Genes

Expression of ZO-1, claudin-1, occludin, nuclear factor-κB (NF-κB), and Nrf2 in the ileal mucosa of weanling pigs were measured by using Western blot analysis according to the previous study [16]. The BCA Protein Assay Kit was used to determine mucosal protein concentration. Extracts containing equal amounts of protein (30 μg) were resolved by 10% polyacrylamide gels and transferred onto PVDF membranes (Millipore, Billerica, MA, USA). The membrane was blocked for 3 h with 5% skimmed milk powder and incubated overnight with antibodies (Abcam, Cambridge, UK) for ZO-1, claudin-1, occludin, NF-κB, Nrf2, and anti-β-tulubin in a 1:2000 dilution. After three washes, the secondary antibody (LI-COR, Nebraska, NE, USA) was added in a 1:10,000 dilution and incubated at 4 °C for 4 h. The membrane was washed three times and developed using WesternBright™ Peroxide (Advansta, San Jose, CA, USA) in an imaging system (Carestream, New York, NY, USA). 

### 2.7. Microbial Community

A Kit (Omega Bio-tek, Norcross, GA, USA) was applied to extract microbial DNA, as described by Zhao et al. [17]. The genes of bacterial 16S ribosomal RNA in the V4-V5 variable region were amplified using PCR with primers. Integrity of PCR amplicons were analyzed by electrophoresis using a Tapestation Instruction (Agilent technologies, Santa Clara, CA, USA). The PCR amplicons were extracted and purified by a DNA Gel Extraction Kit using 2% agarose gels (Axygen Biosciences, Union City, CA, USA). The output was quantified using QuantiFluor™ -ST and sequenced on an Illumina MiSeq system. The QIIME software was used to demultiplex and quality-filter raw Illumina fastq files [18]. The RDP database (http://rdp.cme.msu.edu/; accessed on 28 September 2021) was used to take the taxonomy-based analysis for operational taxonomic units (OTU) using an RDP classifier at a 90% confidence level. To clarify potential interactions between gut microbial community and intestinal barrier functions of piglets, a Spearman correlation analysis was performed to explore the relationship between microbial composition and inflammatory cytokines, host defense peptides, tight junction proteins, or mucins [15].

### 2.8. Production of Lactic Acid and SCFA

Concentrations of lactic acid and SCFA in the ileal and colonic digesta samples were analyzed using methods described by Zhao et al. [19]. Briefly, fecal samples (about 1 g) were put into centrifuge tube (10 mL) and 0.10% hydrochloric acid (2.0 mL) was added. Tubes were placed in an ice bath for 25 min, then mixed and centrifuged at 15,000 rpm to harvest the supernatant. Supernatant was filtered through a 0.45 μm Nylon Membrane Filter (Millipore, Bedford, OH, USA) and then analyzed using a Gas Chromatograph System (Agilent HP 6890 Series, Santa Clara, CA, USA).

### 2.9. Statistical Analysis

A general linear model (GLM) of SAS was used to analyze the observations. Each pen was used for analysis as an experimental unit. Standardized OTU reads were applied to analyze bacterial microbial diversity and composition, which were were analyzed using standardized OTUs reads, according to the procedure of *R* software. The population of the microbial community in intestinal digesta samples of piglets at the phyla, family, and genera levels were analyzed using a Kruskal-Wallis analysis. The differential bacteria were classified using a linear discriminant analysis (LDA) when the logarithmic LDA values of gut microbiota exceeded 2.0. The comparative differences between the two dietary groups was analyzed by using the method of Welch’s *t*-test. Significant differences were considered significant if *p* < 0.05 and as a tendency if 0.05 < *p* < 0.10.

## 3. Results

### 3.1. Effects of FOS Supplementation on Growth Performance and Diarrhea Incidence

**Figure 1:**
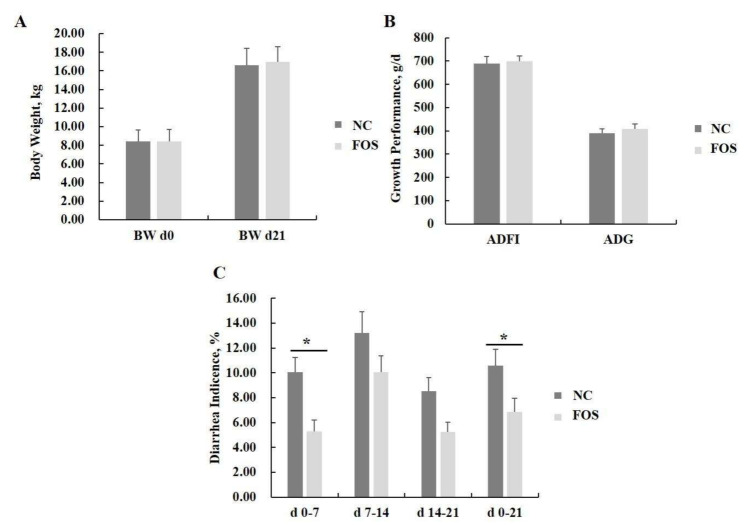
Effects of fructooligosaccharide supplementation on growth performance and diarrhea incidence. (**A**) Pig body weight. (**B**) ADFI and ADG. (**C**) Diarrhea incidence. ADFI, average daily feed intake; ADG, average daily gain; NC, a control diet; FOS, a fructooligosaccharide diet; d, day. * means a significant difference (*p* < 0.05).

Dietary FOS supplementation did not influence the ADFI and ADG of weanling pigs (Figure 1). FOS supplementation significantly decreased incidence of diarrhea from days 0 to 7 and days 0 to 21 of the experiment (*p* < 0.05). In addition, a tendency for FOS supplementation to reduce diarrhea incidence was observed in weanling pigs at days 7–14 and days 14–21 post-weaning (*p* < 0.10).

### 3.2. Effects of FOS Supplementation on Serum Antioxidant Activity, Inflammatory Cytokines, and Intestinal Permeability

**Figure 2:**
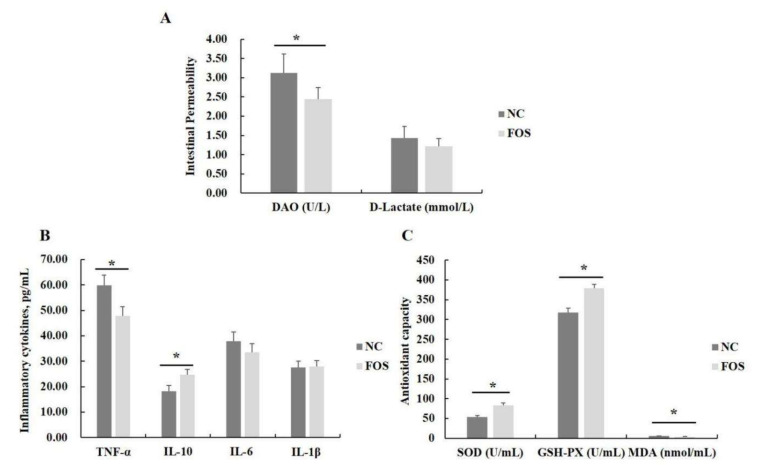
Effects of fructooligosaccharide supplementation on serum parameters. (**A**) DAO and D-lactate. (**B**) Inflammatory cytokines. (**C**) Antioxidant enzymes. DAO, diamine oxidase; IL-1β, interleukin-1β; IL-6, interleukin-6; IL-10, interleukin-10; TNF-α, tumor necrosis factor-α; MDA, malondialdehyde, SOD, superoxide dismutase; GSH-Px, glutathione peroxidase; NC, a control diet; FOS, a fructooligosaccharide diet. * means a significant difference (*p* < 0.05).

The content of DAO in serum of weanling pigs was decreased in the FOS group when compared with the NC group (*p* < 0.05), but FOS supplementation did not influence serum D-lactate concentrations (Figure 2). In addition, FOS supplementation decreased the concentration of TNF-α and increased the content of IL-10 when compared with the NC group (*p* < 0.05), but had no effects on concentrations of IL-6 and IL-β in the serum of weanling pigs. Furthermore, supplementing the diet with 1% FOS improved the activity of SOD and GSH-Px, and reduced serum MDA content in weanling pigs (*p* < 0.05).

### 3.3. Effects of FOS Supplementation on Intestinal Morphology

**Figure 3:**
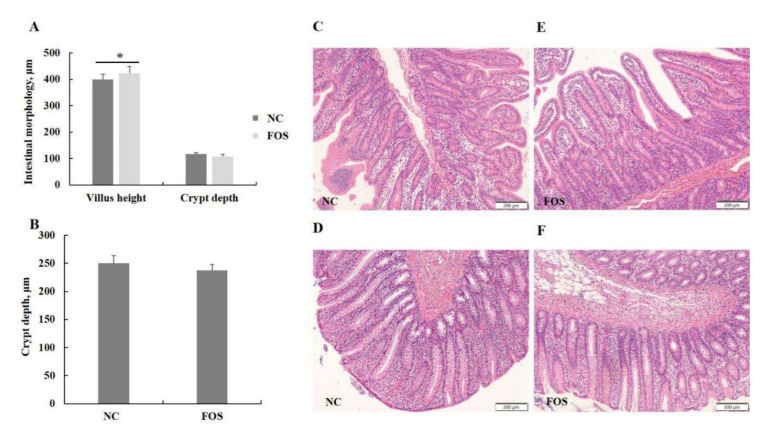
Effects of fructooligosaccharide supplementation on intestinal morphology. (**A**) Ileal morphology. (**B**) Colonic morphology. (**C**) H&E of the ileum in the NC group. (**D**) H&E of the colon in the NC group. (**E**) H&E of the ileum in the FOS group. (**F**) H&E of the colon in the FOS group. NC, a control diet; FOS, a fructooligosaccharide diet. * means a significant difference (*p* < 0.05).

There were no differences in crypt depth in the ileum and colon of weanling pigs between dietary treatments (Figure 3). However, the diet supplemented with 1% FOS increased villus height in the ileum of weanling pigs when compared with the NC group (*p* < 0.05).

### 3.4. Effects of FOS Supplementation on mRNA Expression of Intestinal Inflammatory Cytokines and Host Defense Peptides

**Figure 4:**
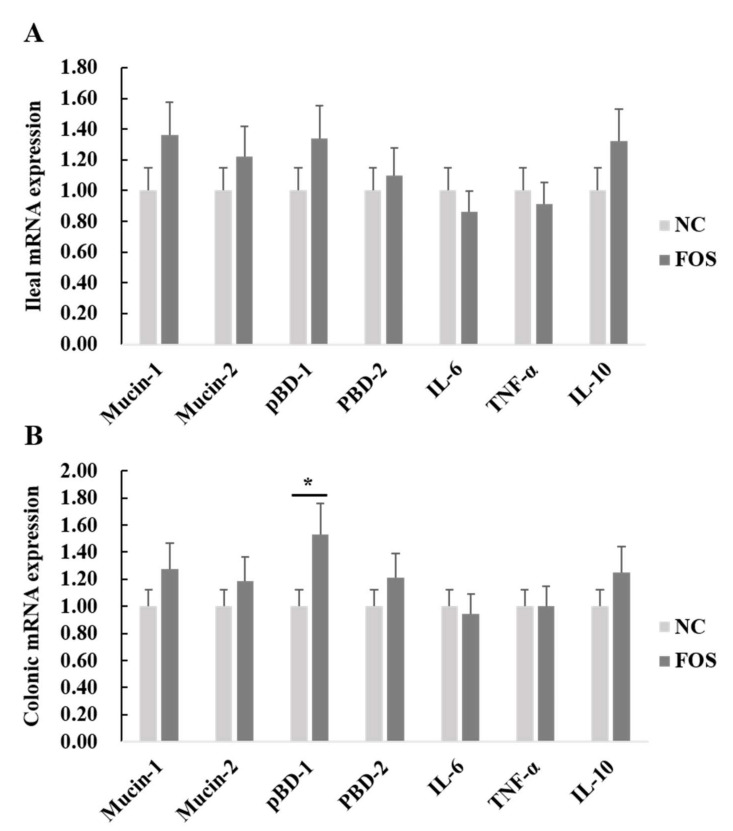
Effects of fructooligosaccharide supplementation on mRNA expression of mucins, host defense peptides, and inflammatory cytokines in the ileum and colon of weanling pigs. (**A**) Ileal mRNA expression. (**B**) Colonic mRNA expression. IL-6, interleukin-6; IL-10, interleukin-10; TNF-α, tumor necrosis factor-α; NC, a control diet; FOS, a fructooligosaccharide diet. * means a significant difference (*p* < 0.05).

The mRNA expression of inflammatory cytokines and host defense peptides in the ileal and colonic mucosa of weanling pigs were detected (Figure 4). There were no differences in the mRNA expression of mucin-1, mucin-2, pBD-1, ZO-1, IL-6, IL-10, or TNF-α in the ileum of weanling pigs between dietary treatments. However, dietary FOS supplementation increased mRNA expression of pBD-1 in the colonic mucosa of weanling pigs (*p* < 0.05).

### 3.5. Effects of FOS Supplementation on Expression of Intestinal Tight Junction Proteins

**Figure 5:**
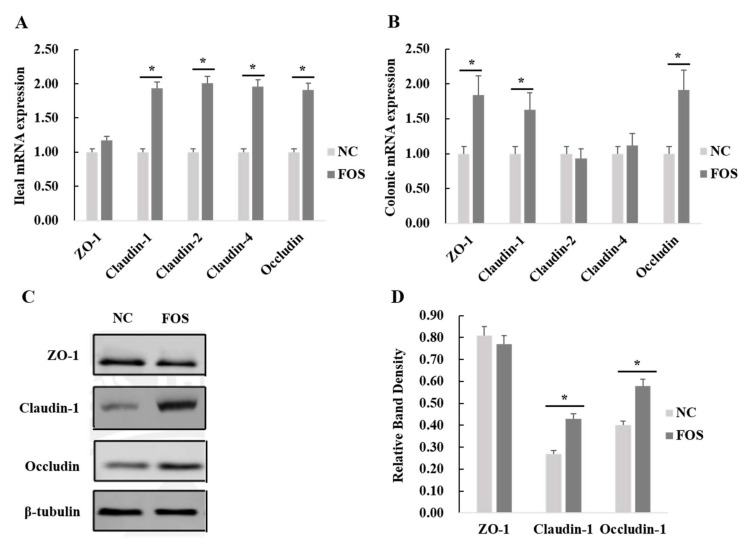
Effects of fructooligosaccharide supplementation on expression of tight junction proteins in the ileum and colon of weanling pigs. (**A**) Ileal mRNA expression. (**B**) Colonic mRNA expression. (**C**) Western Blot for tight junction protein expression in the ileum. (**D**) Relative band density of tight junction protein expression in the ileum. IL-6, interleukin-6; IL-10, interleukin-10; TNF-α, tumor necrosis factor-α; NC, a control diet; FOS, a fructooligosaccharide diet. * means a significant difference (*p* < 0.05).

Expression of tight junction proteins in the ileal and colonic mucosa of weanling pigs was analyzed (Figure 5). FOS supplementation increased mRNA expression of claudin-1, claudin-2, claudin-4, and occludin in the ileal mucosa of weanling pigs (*p* < 0.05). In addition, dietary FOS supplementation significantly increased mRNA expression of ZO-1, claudin-1, occludin, and pBD-1 in the colonic mucosa of weanling pigs (*p* < 0.05).

### 3.6. Effects of FOS Supplementation on Expression of Intestinal Antioxidase Activity

**Figure 6:**
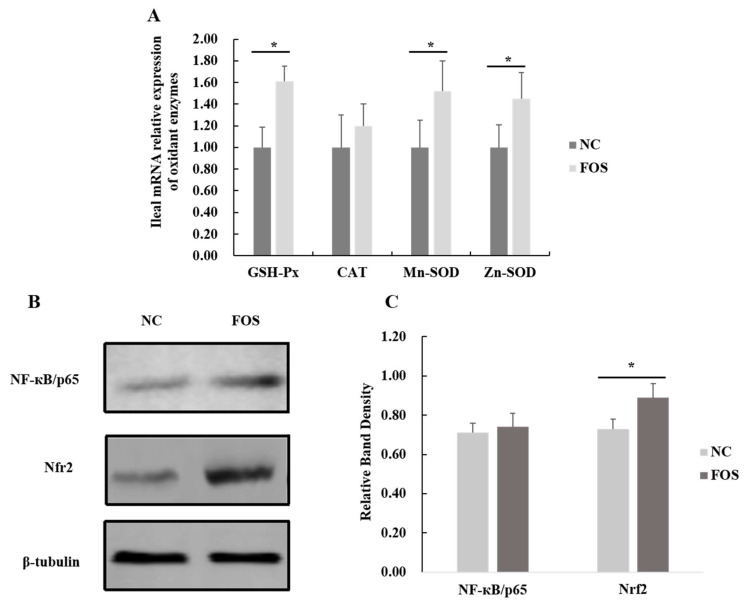
Effects of fructooligosaccharide supplementation on expression of nuclear factor-κB (NF-κB) and nuclear factor erythroid-2 related factor 2 (Nrf2) genes in the ileal mucosa. (**A**) Ileal mRNA expression of antioxidase activity. (**B**) Western Blot for Nrf2 expression. (**C**) Relative band density of Nrf2 expression. NF-κB, nuclear factor-κB; Nrf2, nuclear factor erythroid-2 related factor 2; SOD, superoxide dismutase; GSH-Px, glutathione peroxidase; CAT, catalase; NC, a control diet; FOS, a fructooligosaccharide diet. * means a significant difference (*p* < 0.05).

Dietary FOS supplementation increased mRNA expression of SOD-Mn, SOD-Zn, and GSH-Px in the ileal mucosa of weanling pigs (*p* < 0.05). In addition, expression of Nrf2 in the ileal mucosa of weanling pigs was greater in pigs that were fed dietary FOS when compared with the NC group (*p* < 0.05; Figure 6), and there was a tendency for the decreased expression of NF-κB in samples of the ileal mucosa for the pigs that were fed a FOS diet (*p* = 0.07). 

### 3.7. Effects of FOS Supplementation on Microbial Communities and Their Metabolites

**Figure 7:**
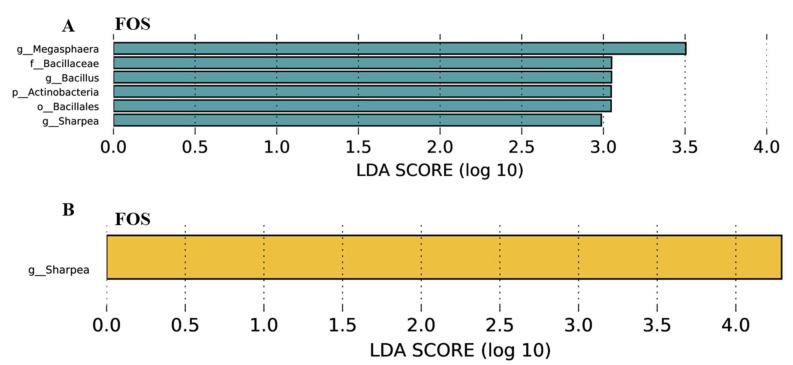
Effects of fructooligosaccharide supplementation on the differential bacteria. (**A**) Differential bacteria in the ileal digesta. (**B**) Differential bacteria in the colonic digesta. Abundances of differential bacteria were classified using linear discriminant analysis (LDA), if the logarithmic LDA values of bacteria exceeded 2.0. The comparative analysis between two dietary groups was conducted by using the method of the Welch’s *t*-test. A value of *n* = 5, 6 individuals per group were kept for the analyses of gut microbiota, but one outlier was eliminated in each group due to the large statistics error. NC, a control diet; FOS, a fructooligosaccharide diet; “g”, “f”, “o”, and “p” represent different taxa of differential bacteria.

Differential microbial communities in the ileal and colonic digesta of weanling pigs between different dietary treatments were analyzed (Supplementary Figure S1). There were no significant differences in microbial α- and β-diversity. Changes of microbial composition shaped by FOS supplementation on phylum and genus levels are shown in Supplementary Figure S2. The differential bacteria in the ileal and colonic digesta of piglets between dietary treatments are presented (Figure 7). In the ileum, dietary supplementation of FOS increased abundance of the phyla Actinobacteria and populations of the genera Megasphaera, Bacillus, and Sharpea (*p* < 0.05). Additionally, in the colon, FOS supplementation in the diet increased abundance of the genera Sharpea (*p* < 0.05).

**Figure 8:**
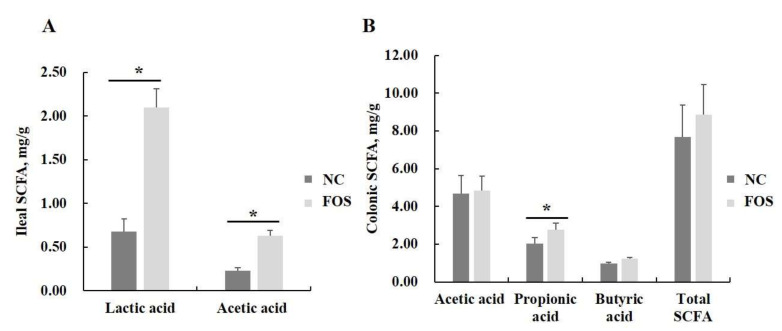
Effects of fructooligosaccharide supplementation on intestinal lactic acid and SCFA concentrations. (**A**) Ileal lactic acid and SCFA. (**B**) Colonic lactic acid and SCFA. NC, control diet; FOS, fructooligosaccharide diet. SCFA, short chain fatty acid. * means a significant difference (*p* < 0.05). Propionic and butyric acid were not detected in ileal digesta, and lactic acid was not detected in the colonic digesta.

Furthermore, FOS supplementation in the diet increased concentrations of lactic acid and acetic acid in ileal digesta and propionic acid in colonic digesta of weanling pigs (*p* < 0.05), but had no significant effects on butyrate concentration in the ileal and colonic digesta of weanling pigs (Figure 8).

### 3.8. Association Analysis between Intestinal Integrity and Microbial Community

**Figure 9:**
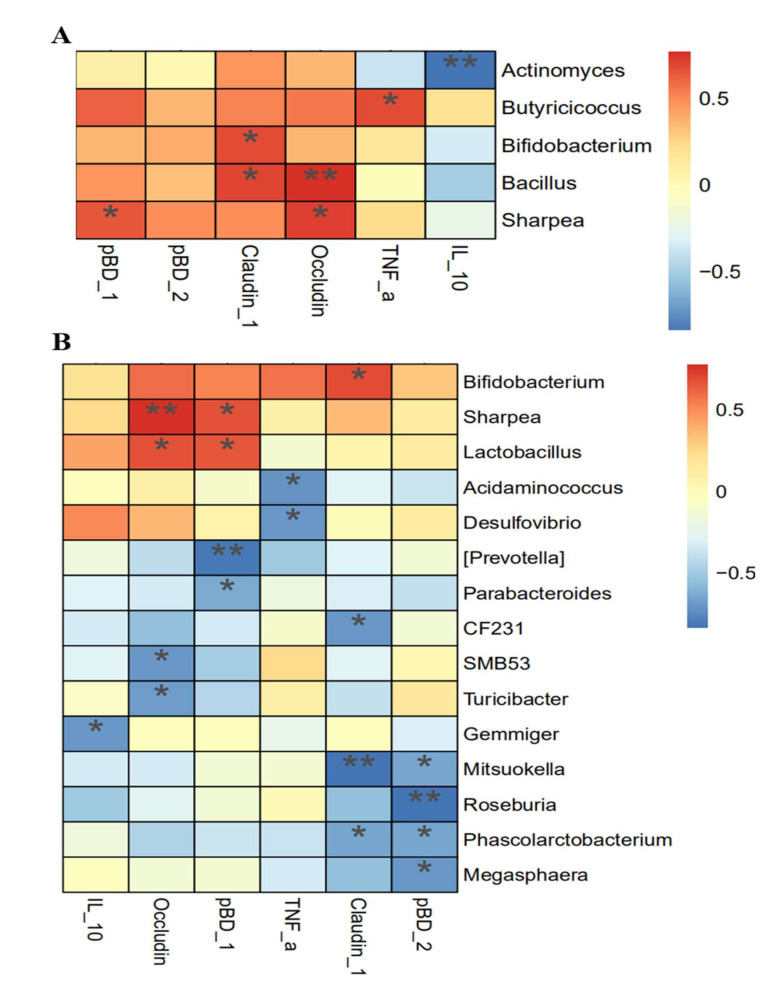
Association analysis between gut microbial composition and intestinal integrity. (**A**) Ileum. (**B**) Colon. IL-10, interleukin-10; TNF-α, tumor necrosis factor-α; NC, a control diet; FOS, a fructooligosaccharide diet. The red and blue color represent positive and negative responses, respectively. *, ** represent *p* < 0.05 and *p* < 0.01.

Association analysis was performed to clarify the potential interactions between intestinal integrity and microbial communities (Figure 9). In the ileum, mRNA expression of claudin-1 was correlated positively with abundances of Bifidobacterium and Bacillus (*p* < 0.05).The mRNA expression of occludin was correlated positively with abundances of Sharpea and Bacillus (*p* < 0.05). In addition, mRNA expression of pBD-1 and TNF-α were correlated positively with abundances of Sharpea and Butyricicoccus, respectively (*p* < 0.05), and mRNA expression of IL-10 was correlated negatively with the abundance of Actinomyces (*p* < 0.05). In the colon, mRNA expressions of both occludin and pBD-1 were correlated positively with abundances of Sharpea and Lactobacillus (*p* < 0.05), and mRNA expression of claudin-1 was positively correlated with an abundance of Bifidobacterium (*p* < 0.05).

## 4. Discussion

### 4.1. Responses of FOS Supplementation on Pig Performance and Diarrhea Incidence

In the current study, FOS supplementation decreased the incidence of diarrhea, which agrees with the finding of Xu et al. [20]. Some publications demonstrated that optimized intestinal microbial composition and SCFA production were associated with the reduced diarrhea when weanling pigs were fed the FOS diet [4,5]. The SCFA promotes expression of host defense peptides and regulates the secretion of pro-inflammatory and anti-inflammatory cytokines by activating GPR and inhibiting HDAC activity to suppress the NF-κB signaling pathway, resulting in improved intestinal immune function of weanling pigs [21]. In addition, a report showed FOS increased the activity of endogenous digestive enzymes, and decreased the fermentation of substrates by harmful bacteria that produce endotoxins in the hindgut, which contributed to the reduce diarrhea [22]. Generally, the reduced diarrhea incidence of weanling pigs treated by FOS is a clinical behavior, which would be caused by complex and systematic interactions among dietary treatment, feeding environment, and pig health. A previous report showed positive effects of the prebiotics on growth performance and intestinal physiological functions were more efficient in a dirty feeding condition when compared with a clean feeding environment [23]. However, no obvious improvement in the growth performance of weaned pigs fed in a comfortable environment was observed in the current study. Thus, the different responses of FOS treatment on growth performance when compared with other publications should be associated with the feeding environment and healthy status of weanling pigs

### 4.2. Responses of Antioxidase Activity and Intestinal Permeability to FOS Supplementation

Diamine oxidase is originally located in epithelial cells to protect biological functions of the intestinal mucosa by promoting cell repair, regulating cellular ion equilibrium, and acting on the transduction pathway of signals [24]. D-lactate is a fermentation metabolite of intestinal microbiota and is rarely absorbed from the integrated intestine. Mammals, such as pigs, cannot secrete endogenous digestive enzymes to degrade D-lactate [25]. However, both DOA and D-lactate can be absorbed easily into the blood when the intestinal barrier function of pigs is damaged. Consequently, the presence of those biological markers in systemic blood reflects intestinal barrier dysfunction [26]. The increased concentrations of DAO and D-lactic acid reflects the damage to the intestinal function of piglets when compared with the NC diet [27]. The lower content of DAO in serum induced by FOS supplementation represented improved intestinal integrity, which contributed to the reduced pig diarrhea in the current study. There are few previous publications reporting the effects of FOS treatment on serum concentrations of DAO and D-lactate; however, a previous report showed that other dietary oligosaccharides, such as alginate-oligosaccharide and chito-oligosaccharide, decreased concentrations of DAO and D-lactic acid in the serum of weanling pigs [28]. The positive response of dietary FOS supplementation on the reduced DAO content is likely related to increased lactic acid and SCFA concentrations in the ileal and colonic of weanling pigs, because lactic acid reduces the intestinal pH to suppress the growth of pathogens, and SCFA serves as an energy source for epithelial cells to improve the intestinal development of weanling pigs [29].

Piglets at weaning would suffer severe oxidative stress, resulting in increased concentrations of reactive oxygen species (ROS) in the intestine, and damage to the intestinal barrier function of piglets [30]. Intestinal epithelial integrity and biological function are closely related to the oxidative status of intestinal epithelial cells [31]. Supplementing diets with 1% FOS improved the activity of SOD and GSH-Px, and reduced serum concentrations of MDA in weanling pigs, which indicated that FOS supplementation partially releases oxidation stress caused by the weaning and is beneficial to gut homeostasis. Many publications reported that functional oligosaccharides serving as prebiotics increase mRNA expression of SOD and GSH-Px in the jejunum of piglets, leading to improved anti-oxidation status of the intestinal mucosa [32,33,34]. To further clarify whether FOS treatment improves antioxidant capacity of weanling pigs by activating Nrf2 signaling, expression of Nrf2 was measured in the present study. Our finding verified the hypothesis that FOS administration increased expression of the Nrf2 gene, which was associated with the improved intestinal anti-oxidase activity in weanling pigs.

### 4.3. Effects of FOS Supplementation on Intestinal Tight Junction Protein Expression

A mixture of short-chain and long-chain FOS, supplemented by using intragastric administration, improved the expression of tight junction proteins in the jejunum of suckling piglets [35]. In the present study, FOS supplementation increased ileal and colonic mRNA expression of tight junction proteins in weanling pigs. The SCFA produced by microbial fermentation of dietary FOS would reduce pH of the intestine and decrease colonization of harmful bacteria, such as *Escherichia coli*, resulting in reduced damage to the intestine [36]. Therefore, the improved expression of tight junction proteins in weanling pigs could be related to an increase in lactic acid and acetic acid concentrations in the ileal digesta of pigs, resulting in an improved intestinal micro-environment. In addition, no significant differences in mRNA expression of pro-inflammatory and anti-inflammatory cytokines after FOS supplementation in the ileal and colonic mucosa of weanling pigs were observed, but FOS supplementation significantly decreased TNF-α concentrations and increased IL-10 content in serum. Previous studies have reported that SCFA produced by the microbial fermentation of FOS could activate GPR and suppress HDAC to down-regulate the expression of NF-κB, resulting in a reduced secretion of pro-inflammatory cytokines [1]. In addition, some reports showed that an increased expression of host defense peptides could be induced by produced SCFA to improve the intestinal immune function of piglets through suppressing the signaling pathways of NF-κB and mitogen-activated protein kinase (MAPK) [37]. Thus, FOS treatment suppressed the expression of NF-κB in the ileal mucosa and increased mRNA expression of pBD-1 in the colonic mucosa of weanling pigs, which possibly was regulated by a greater SCFA production, caused by FOS fermentation.

### 4.4. Effects of FOS Supplementation on Microbial Composition and Their Metabolites

Previous researchers have reported that dietary supplementation with FOS is beneficial to the structure of microbial communities in the intestine of weanling pigs, through promoting the growth of Lactobacillus and Bifidobacterium [38]. Fructooligosaccharides improve microbial composition by providing fermentation substrates for gut bacteria to produce SCFA. In the current study, there were no significant differences in microbial α- and β-diversity in the ileum and colon of weanling pigs. However, FOS supplementation increased the relative abundance of Actinobacteria and populations of the genera Megasphaera, Bacillus, and Sharpea in the ileum, and meanwhile increased the abundance of the genera Sharpea in the colon of weanling pigs. Previous researchers reported fiber inclusion, such as corn bran and wheat bran rich in arabinoxylan, increased the relative abundance of Megasphaera, which can convert lactic acid into propionic and acetic acids [39,40]. Many researchers reported that Bacillus species, such as Bacillus subtilis and Bacillus licheniformis, serving as probiotics, are used to improve the ADG and feed efficiency of piglets. Zhao et al. reported that a diet supplemented with 5% corn bran increased the populations of Bacillus in fecal samples of weanling pigs [17]. Many publications reported FOS supplementation increased the abundance of Lactobacillus and Bifidobactrium in the intestine, but our study did not observe the similar results mentioned above. However, Sharpea is an important lactic acid-producing bacteria in the intestine of young animals [39]. A greater abundance of Sharpea in the FOS when compared with the control groups was consistent with the observation that FOS administration increased lactic acid concentration in the ileal digesta of weanling pigs. In addition, intestinal microbiota composition represents an important defense barrier against oxidation stress from changes in diet and feeding environment for pigs at weaning, and is also a target of ROS damage. The suppression of anti-oxidases induced by ROS damage to intestinal barrier leads to imbalance of gut microbiota composition, which allows harmful bacteria to compete with the beneficial microbiota [41]. The improved anti-oxidase activity in the FOS treatment optimize gut microbial community, such as the presence of increased lactic acid-producing bacteria. A previous report showed that oxidative stress was directly correlated with some gut microbiota; for example, Actinomyces was negatively correlated with ROS concentration in the colon of mice [42]. This demonstrates that FOS modulating the gut microbiota is linked to the antioxidant status of the pigs, however, this mechanism needs to be further investigated.

Short-chain FOS supplemented in the diet of suckling piglets increased concentrations of acetic acid and butyric acid in the cecum [4]. However, in the current study, FOS provided to weanling pigs increased lactic acid and acetic acid concentrations in the ileum, but did not affect concentrations of acetic acid, butyric acid, or total SCFA. Consistent with our findings, previous researchers reported short-chain FOS had positive responses on the concentration of butyric acid in the jejunum and ileum of weanling pigs, but did not affect SCFA concentration in the colon [43], because FOS could be fully fermented by microbiota in the upper gut of piglets due to the molecular structure [44]. In addition, the increased SCFA concentration in the pig’ intestine treated by FOS supplementation is associated with the shaped microbial community, which would increase fermentation of other dietary fiber components.

### 4.5. Association between Intestinal Function and Microbial Communities

Microbial communities in the pig intestine is a complex and dynamic ecosystem that produces crucial metabolites, which always play important roles in maintaining intestinal morphology, facilitating immunological function, and modulating gene expression related to host metabolism [45,46,47]. A previous report showed that a combination of galactooligosaccharide (GOS) and FOS can be utilized by gut microbiota, and their metabolites may enhance the status of intestinal health [48]. In the current study, mRNA expression of tight junction proteins was correlated positively with abundances of Bifidobacterium, Bacillus, and Sharpea, and mRNA expression of pBD-1 was correlated positively with abundances of Sharpea in the ileum of weanling pigs. In the colon, mRNA expressions of tight junction proteins and pBD-1 were correlated positively with abundances of Sharpea, Lactobacillus, and Bifidobacterium. Those results mentioned above indicated that FOS administration is beneficial to intestinal function, which is associated with an increase in lactic acid-producing bacteria. Wu et al. showed that enriched Lactobacillus in new-born piglets on day eight, after a short administration of combined functional oligosaccharides, was positively related with gene expression of TNF-α, claudin-1, and IL-1β, whereas the genus unclassified_f_Lachnospiraceae was negatively associated with gene expressions of ZO-1, TNF-α, and mucin-1 [15]. In addition, metabolites of colonized bacteria would influence the physiological and homeostatic status of the host by activating the Nrf2 signaling pathway to increase the antioxidant capacity of intestinal epithelial cells [49], which is consistent with our findings that FOS treatment improved the activity of SOD and GSH-Px in the serum and intestine of weanling pigs. Therefore, the effects of FOS administration on improvements of intestinal health status are associated with the improved antioxidant capacity of the host, as well as optimizing gut microbial composition and greater SCFA production.

## 5. Conclusions

Dietary supplementation with FOS improved the antioxidant capacity and gene expression of Nrf2 in weanling pigs, as well as increased the abundance of lactic acid-producing bacteria and improved intestinal tight junction protein expression, which were associated with reduced pig diarrhea. Improved expression of tight junction protein is associated with increased abundance of lactic acid-producing bacteria in the intestine of weanling pigs. Studies on how to shape the targeted microbiota in the intestine of weanling pigs by FOS administration to improve intestinal function need to be conducted further.

## Data Availability

Not applicable.

## Supplementary Materials

The following supporting information can be downloaded at: https://www.mdpi.com/article/10.3390/nu14030512/s1, Figure S1: The α-and β-diversity of gut microbial community; Table S1: Ingredients composition and nutritional concentrations of the diets (%, as-fed basis); Table S2: All the primers chosen to study the expression of genes related to intestinal barrier functions in weanling pigs; Figure S2: Microbial composition on the genus and phylum levels.

## Abbreviations

ADFI	average daily feed intake
ADG	average daily gain
FOS	fructooligosaccharide
HDAC	histone deacetylase
IL-1β	interleukin-1β
IL-6	interleukin-6
IL-10	interleukin-10
TNF-α	tumor necrosis factor-α
SCFA	short-chain fatty acids
GPR	G protein-coupled receptors
NF-κB	nuclear factor-κB
DAO	diamine oxidase
MDA	malondialdehyde
SOD	superoxide dismutase
GSH-Px	glutathione peroxidase
OTU	operational taxonomic units
LDA	linear discriminant analysis
MAPK	mitogen-activated protein kinase
