# Supplementary material for: Fructooligosaccharide Reduces Weanling Pig Diarrhea in Conjunction with Improving Intestinal Antioxidase Activity and Tight Junction Protein Expression
Source: Nutrients. 2022 Jan 25;14(3):512. doi: 10.3390/nu14030512 (PMC8838560; doi:10.3390/nu14030512)
Supplement: Supplementary file 1 [file nutrients-14-00512-s001.zip › nutrients-1570159-supplementary.pdf]

Supplementary Table S1. Ingredients composition and nutritional concentrations of the diets (%, as-fed basis)

Ingredients	Dietary treatment	
	CON	FOS
Maize	62.35	61.35
Soybean meal	13.00	13.00
Fructooligosaccharide	0.00	1.00
Extruded full-fat soy	4.00	4.00
Fish meal	2.00	2.00
Whey powder	6.00	6.00
Soy concentrate protein	6.00	6.00
Soy oil	2.77	2.77
Dicalcium phosphate	1.10	1.10
Limstone	1.03	1.03
NaCl	0.30	0.30
Lys	0.60	0.60
Met	0.12	0.12
Thr	0.20	0.20
Trp	0.03	0.03
Premix	0.50	0.50
Nutritional levels, %		
Digestible energy, Kcal/kg	3,540	3,522
Crude protein	18.50	18.50
SID Lys	1.45	1.45
SID Met	0.39	0.39
SID Thr	0.79	0.79
SID Trp	0.22	0.22
Calcium	0.80	0.80
Total phosphate	0.60	0.60

Premix provided the following per kilogram of feed: vitamin A, 12,000 IU; vitamin D, 2,500 IU; vitamin E, 30 IU; vitamin K, 3 mg; vitamin B12, 12 µg; D-pantothenic acid, 10 mg; nicotinic acid, 40 mg; choline chloride, 400 mg; Mn, 40 mg; Zn, 100 mg; Fe, 90 mg; Cu, 8.8 mg; I, 0.35 mg; Se, 0.3 mg. Lys, lysine; Met, methionine; SID, standardized ileal digestibility; Thr, threonine; Trp, tryptophan; CON, a control diet; FOS, a fructooligosaccharide diet.

Supplementary Table S2. All the primers chosen to study the expression of genes related to intestinal barrier functions in weanling pigs

Target genes	Primer forward/reverse	Primer sequence (5’→3’)
Mucin 1	Forward	GTGCCGACGAAAGAACTG
	Reverse	TGCCAGGTTCGAGTAAGAG
Mucin 2	Forward	CTGTGTGGGGCCTGACAA

	Reverse	AGTGCTTGCAGTCGAACTCA
ZO-1	Forward	GCCATCCACTCCTGCCTAT
	Reverse	CGGGACCTGCTCATAACTTC
Occludin	Forward	CAGCAGCAGTGGTAACTTGG
	Reverse	CAGCAGCAGTGGTAACTTGG
Claudin-1	Forward	AAGGACAAAACCGTGTGGGA
	Reverse	CTCTCCCCACATTCGAGATGATT
Claudin-2	Forward	GCTGGCGAACGAGTTCTTAC
	Reverse	AGATGGCGCTAGATGTCACC
Claudin-4	Forward	TCAGCCCTGACTTTGCGTG
	Reverse	ACCTGTCTGTCCACACCAC
TNF-α	Forward	CCACGCTCTTCTGCCTACTGC
	Reverse	TCGGCTTTGACATTGGCTACAA
IL-1β	Forward	CCGCCAAGATATAACTGAC
	Reverse	GCAGCAACCATGTACCAA
IL-6	Forward	AATGCTCTTCACCTCTCC
	Reverse	CACACTTCTCATACTTCTCAC
IL-10	Forward	ACCTGGTAGAAGTGATGCC
	Reverse	CAAGGAGTTGTTTCCGTTA
pBD-1	Forward	TGCCACAGGTGCCGATCT
	Reverse	CTGTTAGCTGCTTAAGGAATAAAGGC
pBD-2	Forward	CCAGAGGTCCGACCACTACA
	Reverse	GGTCCCTTCAATCCTGTTGAA

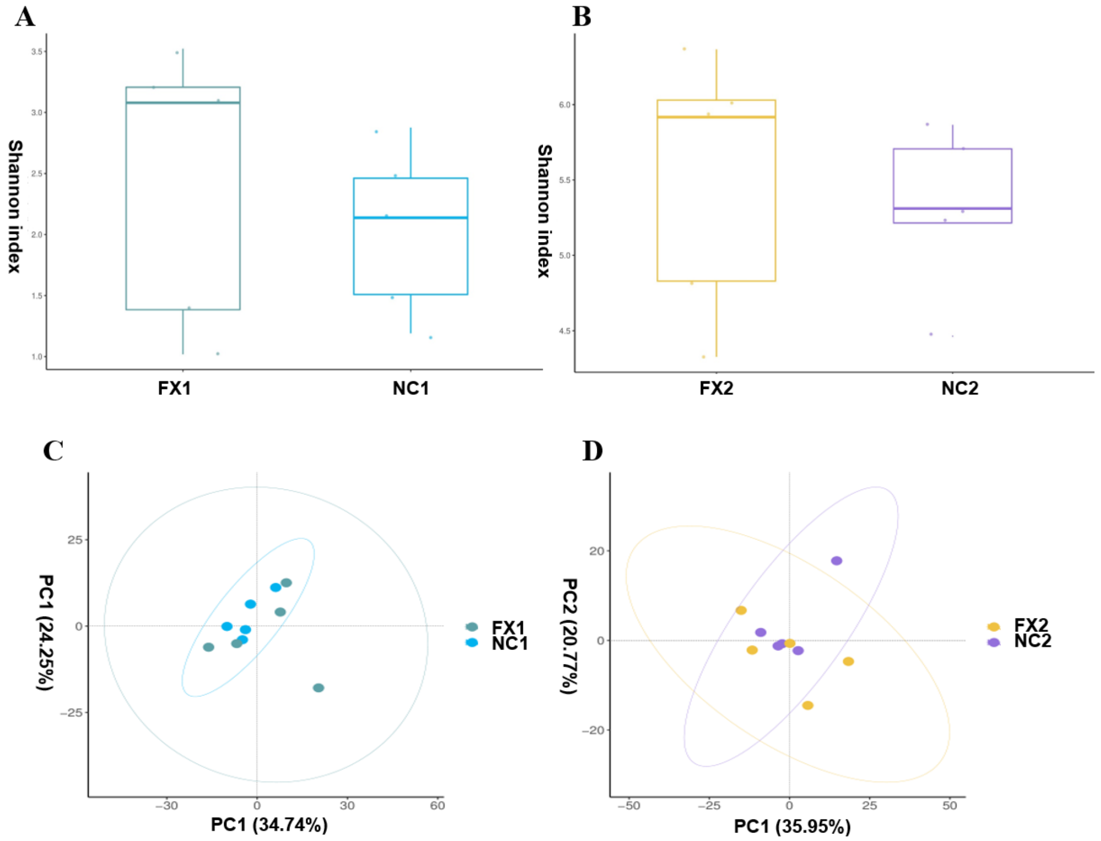

Supplementary Figure S1. The α-and β-diversity of gut microbial community. (A) Microbial α-diversity in the ileal digesta. (B) Microbial α-diversity in the colonic digesta. (C) Microbial β-diversity in the ileal digesta. (D) β-diversity in the colonic digesta. NC, control; FX, fructooligosaccharide. n=5, 6 individuals per group are kept for the analyses of gut microbiota, but one outlier is eliminated in each group due to the big statistics error.

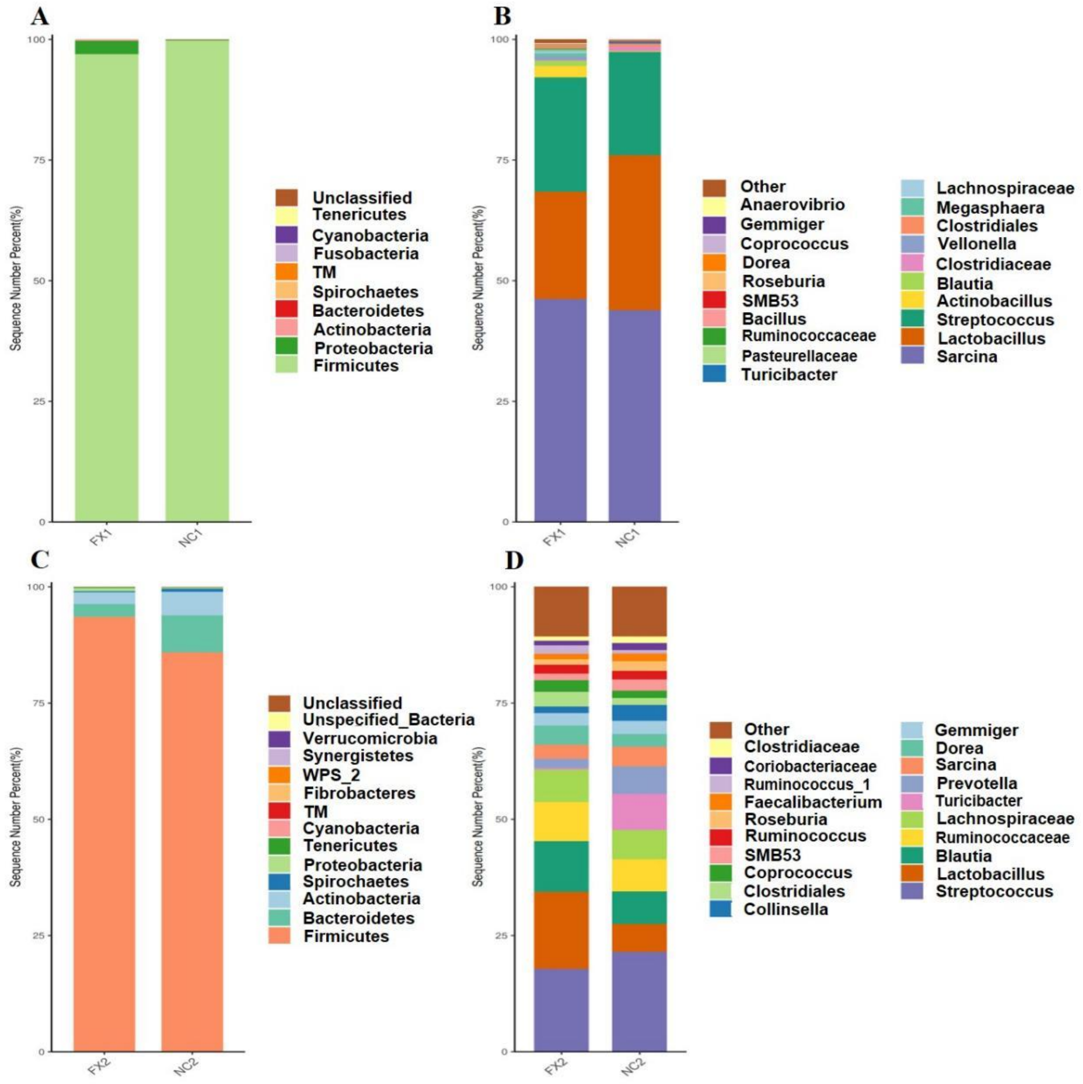

Supplementary Figure S2. Microbial composition on the genus and phylum levels. (A) Ileal microbial composition on the phylum level. (B) Ileal microbial conposition on the genus level. (C) Colonic microbial composition on the phylum level. (B) Colonic microbial composition on the genus level. NC, control; FX, fructooligosaccharide. n=5, 6 individuals per group are kept for the analyses of gut microbiota, but one outlier is eliminated in each group due to the big statistics error.
